# Advances in Calibration Methods for FDR-Based Capacitive Soil Moisture Sensors

**DOI:** 10.3390/s26113366

**Published:** 2026-05-26

**Authors:** Yu Xu, Xizheng Li, Yinghao Song, Yiqi He, Junxiong Peng, Wangling Mei, Kun Zhang, Yuyang Liu, Yue Sun, Xianjun Wu

**Affiliations:** 1 College of Physics and Electronic Engineering, Xinyang Normal University, Xinyang 464300, China; xuyu20084636@126.com (Y.X.); 19513579197@163.com (Y.S.); hyq18@xynu.edu.cn (Y.H.); pengjunxiong0419@163.com (J.P.); mwl24@xynu.edu.cn (W.M.); zk@xynu.edu.cn (K.Z.); yuyangliu@xynu.edu.cn (Y.L.); cathy_sy@126.com (Y.S.); 2 Xinyang Gumai Optronics Co., Ltd., Xinyang 464100, China; xygm88882023@163.com

**Keywords:** capacitive soil moisture sensor, temperature calibration model, conductivity calibration model, Frequency Domain Reflectometry

## Abstract

Soil moisture content plays a crucial role in precision agriculture and geological hazard monitoring, driving the need for stable, reliable, and high-precision sensors. Capacitive soil moisture sensors based on Frequency Domain Reflectometry (FDR) are widely adopted due to their favorable measurement performance, yet their accuracy is highly susceptible to environmental interferences such as temperature, salinity (electrical conductivity), and soil type. This paper systematically reviews current calibration strategies addressing these three factors, classifying them into hardware-based compensation and software-based calibration (including conventional mathematical and machine learning models). Furthermore, it critically analyzes the trade-offs of these approaches in terms of robustness, scalability, and field applicability. To break through current technical limitations, this review argues that future research must prioritize the physical decoupling of multi-parameter interferences under extreme conditions. Additionally, to overcome the generalization crisis of current data-driven models, adaptive strategies utilizing techniques like transfer learning are essential. Finally, implementing Edge-AI on resource-constrained hardware is crucial for achieving calibration-free or real-time online calibration strategies, ensuring long-term measurement accuracy.

## 1. Introduction

Soil moisture plays a crucial role in precision agriculture and geohazard monitoring [1,2]. Researchers have developed various methods for measuring soil moisture, including the gravimetric method, tensiometer method, neutron probe technique, Time Domain Reflectometry (TDR), and Frequency Domain Reflectometry (FDR) [3]. The gravimetric method is regarded as a reference technique. However, it requires laboratory conditions and cannot be monitored in situ. The tensiometer method enables real-time, in situ monitoring, but it cannot be used to directly measure the real water content of soil and is unsuitable for extremely dry soils [3]. The neutron probe technique is unaffected by the physical state of soil water and allows relatively straightforward operation, yet its use is restricted due to radiation risk [3,4,5]. To overcome these limitations, dielectric-based soil moisture sensors have been widely adopted in recent years. Such sensors primarily include TDR- and FDR-based techniques. TDR enables accurate in situ measurement but experiences significant errors in high-salinity soils, and the equipment is costly to acquire and maintain [5]. In contrast, FDR-based methods provide continuous, automatic, and in situ monitoring with no radiation risk, while offering a wide measurement range and high accuracy. These advantages have led to their broad application in both research and practice [3,5].

However, the measurement accuracy of FDR-based soil moisture sensors is influenced by various factors, among which environmental conditions are particularly critical. Numerous studies have demonstrated that soil temperature, salinity, and soil type can significantly affect the performance of FDR capacitive sensors [6,7,8,9,10].

The present review is centered on FDR-based capacitive soil moisture sensors, but it also includes selected studies from other capacitive-sensing applications (e.g., grains or related dielectric media) when they share the same sensing principle—namely, using the measured material as the dielectric medium and inferring target properties from capacitance or dielectric variation—and when their compensation mechanisms are directly transferable to FDR-based soil moisture sensors. Such cross-domain studies are used as methodological support rather than as evidence replacing the soil-sensor literature.

## 2. Methods

### Review Scope, Temporal Scope, and Article Selection

This review was conducted as a narrative literature review with a systematic search and screening procedure. The methodological scope was limited to calibration and compensation studies relevant to FDR-based capacitive soil moisture sensors. Because the objective was to synthesize calibration logic rather than to conduct a meta-analysis, papers were not pooled quantitatively; instead, they were grouped by the principal interference factors and by calibration strategy.

The primary temporal window was defined as 2007–2025. The starting year was selected because the literature search identified 2007 as the earliest point at which BP neural-network-based temperature calibration for capacitive/FDR-related moisture sensing began to appear within the scope of this review. This starting point, therefore, captures the emergence of data-driven temperature-compensation research, while also covering the subsequent development of conductivity, soil-type, and multi-parameter calibration strategies. The endpoint of 2025 was selected to include the most recent studies available at the time of revision, including machine-learning and IoT-oriented calibration strategies. Earlier foundational studies were retained only when they were necessary to explain the dielectric/conductivity principles underlying FDR measurement; they were not used to characterize the recent development trend.

The literature search was performed in the Web of Science Core Collection, IEEE Xplore, ScienceDirect, and CNKI. The search combined the terms “FDR soil moisture sensor”, “capacitive soil moisture sensor”, “temperature compensation”, “temperature calibration”, “salinity calibration”, “salinity compensation”, “electrical conductivity calibration”, “electrical conductivity compensation”, “soil texture calibration”, “soil type calibration”, and “multi-parameter calibration”.

Duplicate records were identified and removed before screening. Records were then screened by title/abstract and, where necessary, by full text. In total, 65 records were retained for the final narrative synthesis.

Studies were included if they met the following criteria: (1) they directly investigated FDR-based or capacitance-based soil moisture sensors; (2) they proposed or evaluated a temperature, electrical-conductivity/salinity, soil-type/texture, or multi-parameter calibration or compensation method; (3) they reported sufficient experimental or methodological information to support comparative synthesis; or (4) they involved another capacitive sensing platform governed by the same dielectric-to-capacitance measurement principle and provided a compensation mechanism transferable to FDR-based soil moisture sensors.

Studies were excluded if they focused exclusively on non-capacitive techniques, discussed soil moisture monitoring without a calibration or compensation target, lacked sufficient methodological detail, were duplicate records, or were unrelated to temperature, conductivity, salinity, soil texture, soil type, or multi-parameter sensor drift.

In terms of manuscript organization, Section 4, Section 5 and Section 6 are arranged first by the three principal interference factors (temperature, electrical conductivity, and soil type) to summarize the dominant calibration strategies under a single-factor perspective, followed by Section 7, which integrates their coupled effects and clarifies why holistic multi-parameter calibration is necessary for realistic FDR deployment.

## 3. Measurement Principles of FDR Soil Moisture Sensors

### 3.1. Dielectric Properties of Soil

The dielectric constant $\varepsilon_{r}$ of soil is used to describe the polarization ability of soil under the action of an electromagnetic field. In general, $\varepsilon_{r}$ is a complex quantity [11] (as shown in Equation (1)). The real part $\varepsilon_{r}^{\prime }$ reflects the soil’s polarization ability, or capacitive property, which is mainly influenced by moisture content and temperature. The imaginary part $\varepsilon_{r}^{\prime \prime }$ represents dielectric loss, which is typically affected by soil salinity, texture, and the measurement frequency.

(1) $$\varepsilon_{r}=\varepsilon_{r}^{\prime }+j\varepsilon_{r}^{\prime \prime }$$

Soil is a mixture composed of solid particles, air, and water. As shown in Table 1, the relative dielectric constant of water (approximately 80) is significantly higher than that of dry soil (2~5) and air. Consequently, soil moisture content can be indirectly estimated by measuring the relative dielectric constant of the soil [12].

### 3.2. Composition and Measurement Principle of FDR Capacitive Soil Moisture Sensor

**Figure 1:**
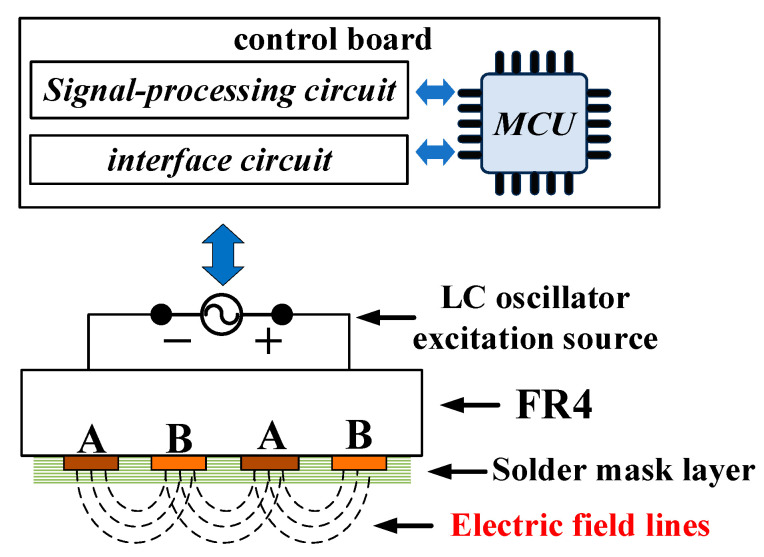
The composition and principle of a capacitance soil moisture sensor. (Adapted from Reference [14]).

FDR-based capacitive soil moisture sensors (hereinafter referred to as ‘capacitive soil moisture sensors’) primarily consist of electrode probes and a high-frequency oscillation circuit. The electrode probe can be regarded as a capacitor using the soil as its dielectric medium [13]. The measurement principle of a capacitive soil moisture sensor is that the soil is used as the medium between the electrode probes (as shown in Figure 1), and the resonant inductance is added to form a resonant circuit with the capacitive sensor. The resonance frequency of this circuit is then measured to determine the soil’s dielectric constant, from which a mathematical model is established to relate the dielectric constant to soil moisture content [14,15,16]. However, as mentioned above, the dielectric constant of soil is affected by environmental factors such as temperature, salinity and soil texture, and this capacitive sensor characterizes soil moisture content by measuring the dielectric constant of soil. To address this issue, various calibration models have been proposed to compensate for the effects of these factors on soil moisture measurements.

**Figure 2:**
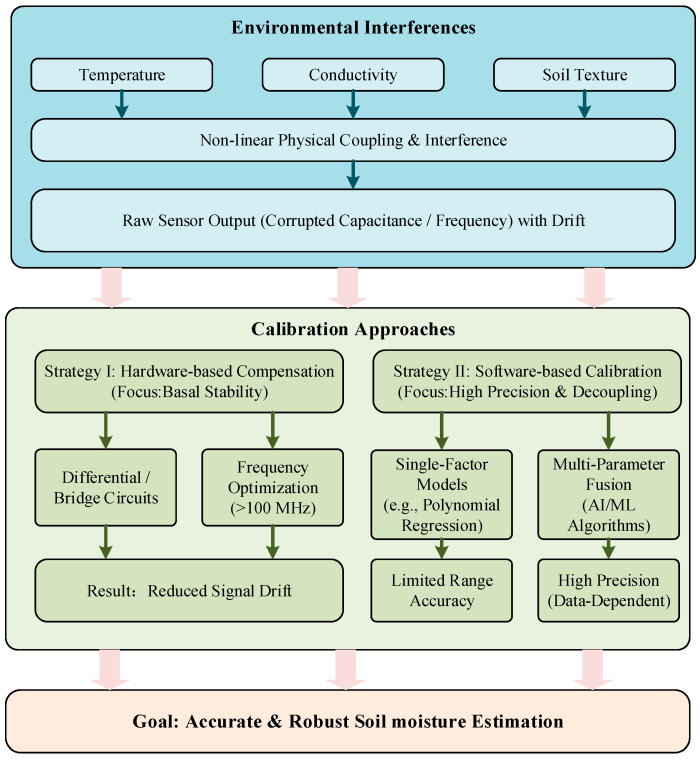
Conceptual framework of environmental interferences and calibration strategies for FDR sensors.

To provide a comprehensive overview of these methodologies, we present a conceptual framework (Figure 2) that illustrates the complete pathway from environmental interferences to the final moisture estimation. As shown in the framework, temperature, conductivity, and soil texture introduce non-linear physical coupling and interference, leading to corrupted raw sensor outputs and signal drift. To mitigate this, current research primarily branches into two strategies: Strategy I (hardware-based compensation) focuses on basal stability through circuit design and frequency optimization, while Strategy II (software-based calibration) aims for high precision and decoupling through mathematical modeling and multi-parameter AI algorithms. This schematic serves as a structural roadmap for the detailed evaluations of each specific calibration strategy in the subsequent sections.

## 4. Recent Advances in Temperature Calibration Methods for Capacitive Soil Moisture Sensors

Capacitive soil moisture sensors are highly sensitive to soil temperature [17,18]. From a dielectric perspective, temperature impacts the sensor mechanism through two opposing physical processes. First, as temperature rises, the thermal agitation of water molecules increases, hindering their alignment with the external electric field; this creates a negative temperature coefficient for pure water dynamics. However, in soil–water–air systems, a second mechanism often dominates: the increased mobility of ions and the release of bound water. Higher temperatures enhance the interfacial polarization (Maxwell–Wagner effect) at the boundary between soil particles and pore water, leading to a significant increase in the apparent dielectric constant, particularly in low-frequency FDR sensors (<100 MHz). Consequently, calibration models must compensate for this nonlinear “fictitious” increase in capacitance caused by thermal ionic activity rather than actual moisture change.

According to previous studies, temperature calibration methods for capacitive soil moisture sensors are generally classified into two categories: hardware-based compensation approaches and software-based calibration strategies. This section mainly discusses the influence of temperature on the output of the sensor and the corresponding calibration method. It should be emphasized that the cross-domain literature cited in this section is limited to the topic of temperature calibration. These studies were not included because they are directly equivalent to soil-moisture experiments, but because they share the same capacitive/FDR-based sensing principle while differing mainly in the measured material or matrix. Therefore, they are used here as methodological references for transferable compensation strategies under a common temperature-drift mechanism, whereas direct quantitative comparison must still rely primarily on soil-based studies. The effects of electrical conductivity and soil type will be discussed in subsequent chapters. Although these factors interact, we will first analyze them from a single-factor perspective, and then summarize their coupling effects in the multi-factor calibration section.

### 4.1. Hardware-Based Temperature Compensation Methods

Hardware-based temperature compensation refers to the use of circuit design to mitigate or eliminate the influence of ambient temperature on the measurement results of capacitive soil moisture sensors. According to existing studies, the most commonly used hardware approaches include the differential capacitance method and the bridge method.

**Figure 3:**
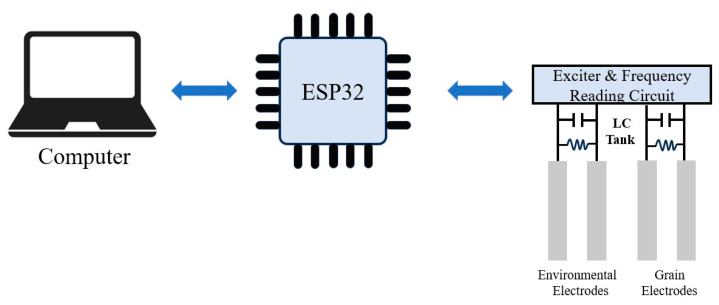
Differential Capacitance Technique (Adapted from Reference [19]).

**Figure 4:**
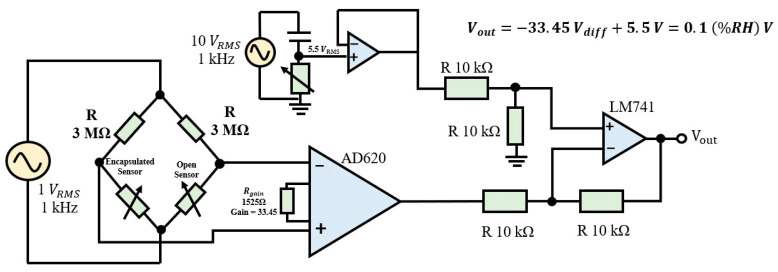
Bridge circuit used for temperature compensation. AD620 denotes the instrumentation amplifier, LM741 denotes the operational amplifier, RH denotes relative humidity, and Vout denotes the output voltage. (Adapted from Reference [20]).

Some representative hardware compensation studies were not initially proposed for soil systems. These studies are discussed here because they share the same basic operating principles, and the temperature drift requiring compensation stems from similar capacitance change mechanisms, despite the different measured media. Therefore, these studies should be considered methodological references for sensor design rather than direct performance benchmarks for soil applications. Mohamed et al. [19] proposed a differential capacitance technique (as shown in Figure 3) to mitigate temperature-induced drift in capacitive sensors. The developed sensor employed a dual-electrode configuration: one set served as the grain-sensing electrodes to measure dielectric variations caused by moisture in grains, while the other set was exposed only to ambient temperature, without contact with the grains, to monitor capacitance drift due to temperature changes. By calculating the difference between the capacitance values measured from the two electrode sets, the temperature effect was effectively compensated. Experimental results demonstrated that temperature-induced capacitance drift was reduced by a factor of four, yielding an improved drift value of 4.9 fF/℃. Javed et al. [20] implemented temperature compensation using a bridge circuit (as shown in Figure 4). Their design incorporates an open sensor and an encapsulated sensor. The open sensor is directly exposed to the air and is affected by both temperature and humidity variations, whereas the encapsulated sensor is isolated from humidity by sealing material, making it responsive only to temperature. By connecting these two sensors to the two opposite arms of the bridge circuit, the differential output signal reflects only humidity changes, thereby achieving effective temperature compensation. Experimental results show that the accuracy of the humidity sensor reached 98.76%, with the temperature-induced error significantly reduced from 22.4% to merely 1.24%.

Despite these designs, hardware-based compensation faces intrinsic physical limitations imposed by circuit characteristics. First, component tolerances and temperature drift are unavoidable. Passive components (resistors and capacitors) in the bridge or LC tank possess their own Temperature Coefficients (e.g., ±50 ppm/℃), making it nearly impossible to perfectly match the thermal response of the reference arm with the sensing probe, leading to residual drift. Second, parasitic effects significantly degrade measurement accuracy. Stray capacitance $\left(C_{stray}\right)$ from PCB traces and connecting cables acts in parallel with the soil sensing capacitance $\left(C_{soil}\right)$. As ambient temperature fluctuates, the dielectric properties of the insulating materials in the circuit board and cables change, introducing a “parasitic drift” that differential circuits often fail to isolate. Finally, the effectiveness of hardware compensation is constrained by frequency dependence. While increasing the operating frequency (e.g., >100 MHz) helps minimize the imaginary part of the dielectric constant (conductivity loss), it exacerbates the influence of parasitic inductance and skin effects, thereby reducing signal stability. Given these insurmountable physical constraints and the inherent bias introduced by electronic component drift [21], recent research has increasingly focused on software-based temperature calibration approaches to achieve higher measurement accuracy.

### 4.2. Software-Based Temperature Calibration Strategies

Software-based temperature calibration models correct the output of capacitive soil moisture sensors using mathematical formulations, thereby compensating for temperature effects. Compared with hardware approaches, software calibration offers greater flexibility and adaptability. Existing software methods can generally be divided into two categories: conventional calibration models, such as linear or polynomial regression, and machine learning–based models.

#### 4.2.1. Conventional Calibration Models

Conventional calibration models use regression to link temperature and sensor readings with actual soil moisture. The least squares method typically determines the regression coefficients [22,23]. However, although these polynomial models are fixed in form and easy to calculate, their ability to characterize complex nonlinear relationships is limited. More importantly, the complexity of the model is difficult to adjust after the polynomial order is selected, which limits its accurate adaptation ability in a changing environment.

#### 4.2.2. Machine Learning-Based Models

Studies have shown that the measurement error of some humidity sensors is linear near room temperature but becomes nonlinear under high- or low-temperature conditions; some sensors also exhibit nonlinearity even around room temperature [24,25]. Traditional multiple regression models, due to their fixed polynomial order, are limited in their capacity to fit such complex nonlinear relationships, thus failing to meet the requirements for high-precision calibration. Therefore, in order to achieve higher calibration accuracy, machine learning models with stronger nonlinear fitting ability are introduced into the temperature calibration research of sensors. In this subsection, several studies from other capacitance-based moisture-sensing applications are also retained because the sensing principle remains the same, whereas the measured material differs. Their relevance lies in providing transferable modeling strategies for temperature compensation under a shared nonlinear drift mechanism, rather than in enabling direct metric-level comparison with soil-moisture studies. From a critical review perspective, however, these ML approaches should not be judged only by their reported $R^{2}$, MSE, or RMSE values. They can be broadly divided into purely data-driven models, which learn statistical mappings directly from sensor outputs and auxiliary variables, and more physically grounded strategies, in which input selection or model design is informed by known dielectric response mechanisms. This distinction is important because a model may appear highly accurate within a controlled dataset but still fail to capture the actual temperature-dependent dielectric behavior of soil [21,26]. From a deployment perspective, however, different ML families also differ substantially in training burden, memory footprint, inference latency, and energy demand, yet these quantities are rarely reported in the reviewed studies, which makes it difficult to judge their suitability for low-power or embedded field nodes from accuracy metrics alone.

**Figure 5:**
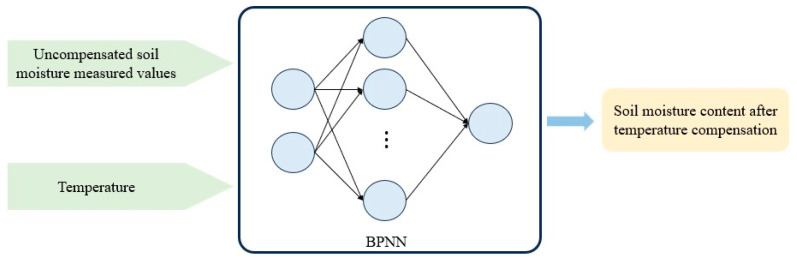
Structure of the BP neural network (BPNN, Back Propagation Neural Network). (Adapted from Reference [27]).

Gnecchi et al. [27] developed a capacitive soil moisture sensor and applied a Back Propagation Neural Network (BPNN) to perform temperature compensation on its output (as shown in Figure 5). Experimental results demonstrated that the sensor readings calibrated with the BPNN were more closely aligned with the gravimetric method and exhibited higher accuracy than those obtained from TDR-based soil moisture sensors. Han et al. [28] developed a dual-capacitance device for measuring corn moisture content. For corn samples with three different porosity levels, they established Support Vector Machine (SVM) regression models to correlate the sensor’s moisture readings with temperature. Experimental results showed that the coefficient of determination ($R^{2}$) for the model exceeded 0.91. In stability tests, the standard deviation was 1.09%. And during dynamic verification tests, the maximum measurement error was 4.62%. Shen et al. [29] proposed a temperature compensation model based on the XGBoost algorithm. They prepared soil samples with varying moisture content (10%~35%) and collected sensor data across a temperature range of 0~45 °C to develop and validate the model. The results demonstrated that the XGBoost model effectively corrected temperature-induced measurement deviations, achieving superior performance on the test set (the MSE, MAE, RMSE and $R^{2}$ of the test set are 0.013%, 0.825%, 1.165% and 0.973) compared to other machine learning methods such as Tree, Random Forest, Bagging, Adaboost, K-Nearest Neighbor (KNN), and Support Vector Regression (SVR) models. Chen et al. [30] applied data-driven models, Gaussian Process Regression (GPR) and Multivariate Adaptive Regression Splines (MARS), to sensor temperature calibration based on FDR technology. The research results show that these two methods have certain advantages in dealing with nonlinear relationships and improving calibration accuracy, showing the application potential in sensor temperature compensation.

While the aforementioned machine learning algorithms have demonstrated significant potential, artificial neural networks (ANNs) remain one of the most extensively adopted architectures in this domain, as they are exceptionally well-suited for modeling highly nonlinear and complex problems. Among them, the backpropagation neural network (BPNN) is particularly popular due to its strong nonlinear fitting capability and ease of implementation. However, the initial weights and biases of BPNNs are typically assigned randomly. When the learning rate is low and the initialization is far from the global optimum, the training process tends to converge to local minima. Moreover, the long training time further increases computational costs. To address these limitations, researchers have explored combining BPNNs with metaheuristic algorithms to alleviate the local optimum problem and enhance training efficiency [31].

Sun et al. [32] employed the capacitance and temperature values from a capacitive rice moisture detection device as input variables to BPNN. A Genetic Algorithm (GA) was used to optimize the network’s weights and biases. The experimental results showed that the mean squared error (MSE) of the GA-BPNN was $1.9683\times 10^{-5}$, which was significantly lower than that of the unoptimized BPNN (MSE = $7.1215\times 10^{-4}$), demonstrating that GA effectively enhanced the prediction accuracy of the BPNN. Jiang et al. [33] integrated measurements from multiple sensors into a BPNN and employed the Whale Optimization Algorithm (WOA) to optimize its weights and biases. The experimental results demonstrated that, compared with traditional machine learning methods such as BPNN, Support Vector Machines (SVM), and Random Forests (RF), the WOA-BPNN model exhibited superior predictive accuracy and stability. Specifically, it achieved a maximum accuracy of 100% and an average accuracy of 98.8%. Qiu et al. [34] developed a differential-capacitance-based device for moisture detection and constructed analytical models of millet moisture content, temperature, and volumetric occupancy using both backpropagation (BP) and Extreme Learning Machine (ELM) algorithms. To improve model performance, the Sparrow Search Algorithm (SSA) was employed to optimize the BP and ELM models. However, due to the reduction in population diversity in later iterations, SSA is prone to problems such as convergence to local optima. To address this issue, a Logistic chaotic map was incorporated to enhance the optimization capability of SSA. Comparative analysis demonstrated that the Logistic-SSA-ELM model achieved superior predictive accuracy, with a prediction set coefficient of determination ($R^{2}$) of 0.7016, a root mean square error of prediction (RMSEP) of 3.7150, and a ratio of performance to deviation of prediction (RPDP) of 1.4035.

To accommodate a broader temperature measurement range, several studies have proposed segmented compensation methods that adjust sensor outputs according to the characteristics of different temperature intervals. Xu et al. [24] developed an adaptive temperature compensation approach for humidity sensors, in which the temperature range was partitioned into a linear interval (22.36 °C~29.98 °C) and nonlinear intervals (<22.36 °C and >29.98 °C) based on the coefficient of determination ($R^{2}$). A least-squares linear fitting was applied within the linear interval, while in the nonlinear intervals, a Genetic Simulated Annealing algorithm (GSA) was employed to optimize the initial weights and biases of a backpropagation neural network. Experimental results demonstrated that this adaptive segmented compensation method significantly reduced measurement errors, thereby improving the accuracy of sensors across a wide temperature range. Xing et al. [25] proposed a segmented calibration strategy based on the residuals of humidity sensor characteristic curves under different temperature conditions. The temperature range was divided into three intervals: −30 °C~0 °C, 0 °C~20 °C, and 20 °C~50 °C. A least-squares linear fitting was applied in the 0 °C~20 °C interval, while Radial Basis Function Neural Networks (RBFNNs) were employed for the −30 °C~0 °C and 20 °C~50 °C intervals. Experimental results demonstrated that, compared with calibration models based solely on BP neural networks or least-squares polynomials, the proposed method achieved faster fitting speed and higher compensation accuracy.

To improve readability, Table 2, Table 3, Table 4, Table 5 and Table 6 are presented as structured evidence summary tables. Although each table uses a uniform reporting structure, the reviewed studies were conducted under significantly different conditions, including sensor type, operating frequency, test medium, environmental range, reference methods, and reporting metrics. Therefore, a rigorous comparison of each study at the metric level is not reasonable from a methodological perspective. The purpose of these tables is to organize the literature according to the same interpretive dimensions, including method category, representative methods and original reported performance metrics.

Table 2 summarizes representative temperature-calibration methods and their within-study effectiveness. For this section, the listed literature includes both direct soil-based studies and a limited number of methodologically relevant studies from other capacitance-based sensing applications. Accordingly, hardware compensation methods are limited by the inherent temperature drift of electronic components, which restricts their compensation accuracy, but they generally demonstrate better stability and ease of use under complex working conditions [35]. In contrast, software compensation methods—particularly machine learning (ML) models—offer greater flexibility and can theoretically achieve full compensation by precisely fitting complex nonlinear relationships.

However, relying heavily on ML models introduces critical risks of over-parameterization and overfitting, alongside high computational complexity and poor interpretability [21,26]. Many of the reviewed ML algorithms are highly parameterized; when trained on limited, laboratory-generated datasets, they tend to memorize specific noise patterns and experimental artifacts rather than learning the underlying dielectric physics. Consequently, their generalization limits are often severely exposed when subjected to dynamic, uncontrolled field conditions. Furthermore, the transferability of these models remains a major challenge. A predictive model optimized for a specific dataset (e.g., a specific soil texture within a narrow temperature range) usually experiences a sharp drop in accuracy when deployed in a new geographical region or different soil type, necessitating complete retraining. Therefore, rather than merely reporting algorithmic performance improvements, future research must rigorously evaluate dataset size, diversity, and employ regularization techniques to prevent overfitting. In addition, most of the reviewed studies do not report computational indicators such as model size, runtime latency, or power consumption, so deployment feasibility cannot be inferred directly from laboratory accuracy alone.

Consequently, hardware compensation remains an ideal choice in applications prioritizing long-term stability and mass deployment with relatively low accuracy requirements. Software compensation, on the other hand, is recommended only in scenarios where sufficient computational resources are available and where the models are trained on adequately large, diverse datasets to ensure true field robustness and high precision. Accordingly, for software-based calibration, model selection should balance nonlinear fitting ability against both generalization performance and deployment cost.

## 5. Research Advances in Conductivity Calibration Models for Capacitive Soil Moisture Sensors

Soil salinity primarily affects the imaginary part of the soil’s relative dielectric constant ($\varepsilon_{r}^{\prime \prime }$) [36]. In capacitive sensors operating at lower frequencies (e.g., tens of MHz), dissolved salts increase electrical conductivity, creating a conductive pathway through the soil matrix. This leads to substantial ionic conduction losses, which dampen the oscillation amplitude or shift the resonant frequency of the LC circuit, mimicking the signal response of higher water content. Physically, this is often described by the loss tangent $tan\delta =\varepsilon_{r}^{\prime \prime }/\varepsilon_{r}^{\prime }$. When salinity is high, the loss tangent increases significantly, causing the sensor to overestimate soil moisture.

Electrical conductivity is widely recognized as a key indicator of soil salinity. As the concentration of dissolved salts in the soil solution increases, the electrical conductivity correspondingly rises. Hence, electrical conductivity measurements provide an effective means of characterizing soil salinity levels [37,38]. This section focuses on the influence of conductivity on the sensor output and the calibration method. There is an interaction between conductivity and temperature, and its combined effect will be further discussed in the multi-factor calibration section.

### 5.1. Mathematical Modeling Approaches for Electrical Conductivity Calibration

To overcome the interference of soil conductivity on water content measurement, researchers usually use model-driven calibration strategies. The mainstream method of this strategy is to construct a mathematical model that integrates the output value of the sensor, the soil conductivity and the real moisture content, and compensates for the measurement deviation accordingly.

To address the limited applicability of calibration models for capacitive soil moisture sensors in saline soils, Zhang et al. [39] proposed a two-step calibration method that accounts for the influence of electrical conductivity on relative permittivity. The first step established a binary quadratic regression model ($R^{2}$ = 0.979) with sensor output voltage and electrical conductivity as inputs and relative soil permittivity as the output. The second step developed a third-order polynomial model (R^2^ = 0.996) relating soil relative permittivity to volumetric water content. Experimental results showed that when the soil electrical conductivity was in the range of 0~2 dS/m, the maximum error of volumetric water content detection after conductivity compensation was reduced to $0.0127\;m^{3}/m^{3}$, and the maximum relative error decreased from 12.0200% to 6.2241%. To improve the measurement accuracy of low-frequency capacitive sensors in complex soils, Deng et al. [38] established the relationship model of soil moisture content, capacitance and electrical conductivity based on the dielectric properties of soil. They systematically compared the fitting effects of three mathematical methods (Logistic, exponential, and polynomial) to identify the optimal compensation model. Experimental results showed that the Logistic-based soil moisture prediction model performed best, with MAME less than 3.55% and MAE below 2.50%.

Several studies have attempted to mitigate the influence of electrical conductivity on soil moisture measurements through hardware design. However, hardware approaches alone are insufficient to completely eliminate electrical conductivity effects. Therefore, mathematical modeling is often employed to further compensate for the measurement deviations caused by electrical conductivity.

**Figure 6:**
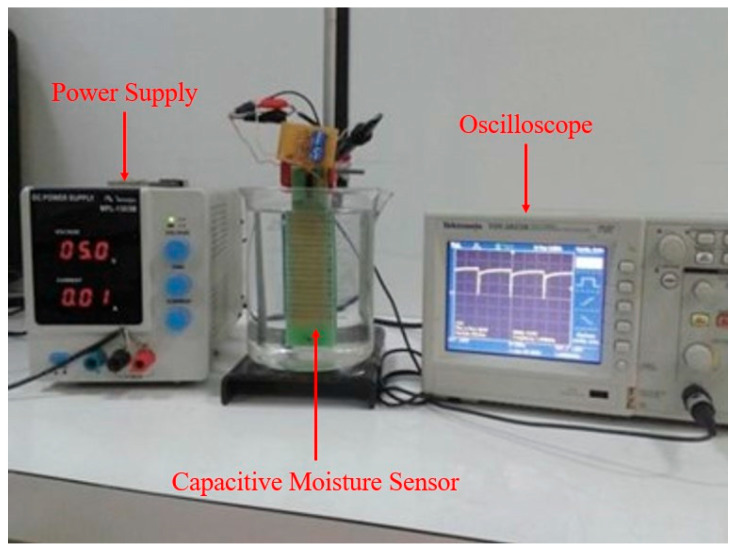
System for measuring the solutions with different levels of salinity using the capacitive sensor (Adapted from Reference [40]).

Santos et al. [40] investigated the performance of a capacitive moisture sensor under different saline conditions and found the sensor to be sensitive to salinity (as shown in Figure 6). They attempted to reduce the interference of salinity on moisture measurements by adjusting the parameters of the RC circuit to increase the operating frequency. The results showed that increasing the measurement frequency helped mitigate the influence of salinity on the sensor readings, but the RC circuit used failed to completely eliminate the interference. Finally, a regression equation ($R^{2}=\;0.9185$) was proposed to describe the relationship between the sensor’s frequency output signal, soil moisture, and salinity.

Overall, Table 3 should be interpreted as a structured summary of conductivity-calibration evidence rather than a direct quantitative comparison. Within that boundary, the literature consistently suggests that hardware/frequency adjustment is simple to implement but has limited compensation capability, whereas mathematical modeling more effectively captures the nonlinear relationship among conductivity, sensor output, and soil moisture content. 

**Table 3:** Structured evidence summary of conductivity-calibration methods.

Category	Reference	Method	Key Performance/Effect	Metrics (Note: Conditions Vary)
Mathematical Modeling	[38]	Logistic Model	Outperformed exponential/polynomial models	MAME < 3.55%; MAE < 2.50%
	[39]	Two-step Regression	Max relative error dropped from 12.02% to 6.22%	$\mathrm{Step}\;1:\;R^{2}$: 0.979 $\mathrm{Step}\;2:\;R^{2}$: 0.996
Hardware/Frequency	[40]	Adjust the frequency of the RC circuit	Frequency increases mitigated salinity impact	$R^{2}$: 0.9185

Note: Table 3 is organized using the same reporting structure as Table 2, but the reported values still come from non-uniform testing conditions. Differences in salinity range, soil, sensor configuration, and validation protocol prevent a strict head-to-head comparison; accordingly, the table should be read as a structured summary of representative evidence rather than a ranking table.

### 5.2. Temperature and Electrical Conductivity Calibration Model

The accurate measurement of soil moisture content faces serious challenges due to the true non-linear coupling mechanisms between temperature (T) and electrical conductivity. To provide a unifying conceptual framework for multi-parameter calibration, this coupling must be analyzed based on the established complex dielectric permittivity defined in Section 3.1 (Equation (1)). According to fundamental electromagnetic theory, the apparent dielectric constant measured by capacitive sensors is governed by both the real permittivity $\left(\varepsilon_{r}^{\prime }\right)$ and the dielectric loss $\left(\varepsilon_{r}^{\prime \prime }\right)$. The true coupling mechanism arises because T simultaneously acts on both components through a dual pathway [17,41,42,43,44]. On one hand, T affects $\varepsilon_{r}^{\prime }$ by altering molecular thermal agitation and interfacial polarization. On the other hand, T fundamentally governs electrical conductivity because higher temperatures decrease pore-water viscosity and significantly enhance ion mobility in the soil solution. Consequently, the ionic conduction loss component of $\varepsilon_{r}^{\prime \prime }$ is mathematically proportional to this temperature-dependent conductivity. Because an increase in temperature inherently magnifies the conductivity-induced dielectric loss, the variables T and electrical conductivity are inextricably cross-coupled. This established physical framework demonstrates that the sensor’s response to environmental changes is a non-linear superposition of these competing effects, rendering simple, isolated linear corrections invalid. Therefore, researchers have proposed a series of multi-parameter mathematical models to fit this complex, cross-coupled relationship to obtain accurate moisture content.

In order to improve the accuracy of the low-cost low-frequency capacitive soil moisture sensor, Gu et al. [45] proposed a multi-parameter correction method. By introducing three parameters of temperature, conductivity and capacitance, a mathematical model for predicting soil moisture was established. The core steps are as follows: first, the measured values of capacitance and conductivity are corrected to the reference temperature (20 °C), and then the capacitance-moisture function and capacitance-conductivity function are fitted, and the compensation model is constructed by using these functions; finally, the corrected soil moisture content is obtained by superimposing the reference moisture value and compensation value. The experimental results show that the maximum measurement error of this method (taking loess as an example) is less than 3.0%, and the average absolute error is less than 2.0%. Bogena et al. [46] corrected the dielectric constant of the EC-5 (METER Group, Pullman, WA, USA) sensor by considering the influence of temperature and conductivity. For the influence of temperature, the author proposes a third-order polynomial temperature correction function with RMSE = 12.20%. For the conductivity deviation, the author uses a fourth-order polynomial correction function with RMSE = 7.22%. These correction terms are added to the original dielectric constant measured by the sensor. The field experiment results show that the measurement deviation is significantly reduced, and the Nash–Sutcliffe efficiency coefficient is increased from 0.23 to 0.74, which verifies the effectiveness of the correction method.

Machine learning technology has been applied to multi-parameter fusion calibration models. To correct for the effects of varying temperature and salinity on the measurement accuracy of the 5TE (METER Group, Pullman, WA, USA) capacitive soil moisture sensor, Wang et al. [47] used the soil dielectric constant, conductivity and temperature data measured by the sensor as the input of the model, and established a soil moisture content prediction model based on Random Forest. The RF model achieved a significantly higher prediction accuracy (RMSE=5%, $R^{2}=0.77$) compared to the modified Topp equation, which relies solely on dielectric permittivity (RMSE=7%, $R^{2}=0.54$). Based on Artificial Neural Network (ANN) technology, Moghadas et al. [48] used electrical conductivity and temperature as the input of the network model to predict soil moisture content. The results show that the ANN-based method is superior to the Rhoades model [49].

Accordingly, Table 4 is used to summarize how different studies handle coupled interference, validation context, and within-study effectiveness, rather than to establish a raw metric ranking. Within this qualitative framework, current research shows that mathematical models remain dominant, but many studies still insufficiently address the full synergistic interference mechanism between temperature and conductivity.

**Table 4:** Structured evidence summary of coupled temperature-conductivity calibration methods.

Category	Reference	Method	Key Performance/Effect	Metrics (Note: Conditions Vary)
Empirical/Mathematical	[45]	Multi-Parameter Correction	Corrected to reference temp (20 °C)	Max Error < 3.0%; Mean Abs Error < 2.0%;
	[46]	Polynomial Correction	Significant improvement in Nash–Sutcliffe efficiency	NSE increased from 0.23 to 0.74;
Data-Driven (ML)	[47]	Random Forest	Superior to modified Topp equation	$R^{2}:\;0.77;\;RMSE:\;5\%$;
	[48]	ANN	Outperformed traditional Rhoades mode	-

Note: Table 4 follows the same reporting logic as Table 2 and Table 3. Because these studies involve coupled temperature-conductivity effects under different soil textures, environmental ranges, sensor types, and evaluation metrics, their reported values are not directly comparable in a strict quantitative sense and should be interpreted within the boundary conditions of each study. “-” indicates data not reported in the original source.

Table 4 lists the comparisons among different calibration methods for multiple parameters. In summary, while constructing mathematical models to compensate for conductivity interference remains the predominant approach in current research, there is a common limitation: the synergistic interference mechanism of temperature and conductivity is ignored. Temperature fluctuation not only affects the soil conductivity, but also significantly affects the real part of the dielectric constant, thus interfering with the accurate measurement of water content. Therefore, in order to develop a soil moisture sensor with high precision and a wide application range, a multi-factor coupling calibration strategy must be adopted, and the interaction of environmental variables such as temperature and conductivity should be considered at the same time. In addition, increasing the operating frequency of the sensor (>500 MHz) can reduce the influence of conductivity on the relative dielectric constant, but too high an operating frequency will not only bring difficulties to the design of the circuit but also increase the cost of the circuit [38,50,51]. Therefore, in practical design, it is necessary to seek the best balance between frequency selection, multi-environmental factor compensation, cost control and system complexity.

In addition, when the capacitive soil moisture sensor is deployed in the field environment for a long time, its measurement results may also be affected by the drift caused by slow environmental changes (such as soil salt accumulation). From the application point of view, in recent years, some studies have tried to use data-driven methods to perform real-time or adaptive calibration of soil moisture sensors. For example, a study used deep neural networks to model the original sensor readings to achieve real-time calibration of the sensor output under field conditions, thereby improving measurement accuracy [52]. Although the related research is still in the exploratory stage, such methods provide new ideas for dealing with problems such as multi-parameter coupling and long-term drift. In the future, a multi-parameter calibration strategy, real-time calibration method and IoT monitoring system [53,54,55] should be combined to realize real-time acquisition, transmission and compensation of sensor data, so as to realize long-term stable and accurate monitoring under multi-environmental parameters.

## 6. Research Progress on Soil-Type Calibration Models for Capacitive Soil Moisture Sensors

Due to the heterogeneity and structural differences in soil, different types of soil will affect the sensitivity of capacitive sensors [56]. This heterogeneity affects sensor sensitivity primarily through the ratio of bound water to free water. Soil particles, particularly clay, possess large specific surface areas and surface charges that tightly bind water molecules, forming a “bound water” layer. These bound water molecules have restricted rotational freedom compared to “free water” in bulk pores, resulting in a significantly lower dielectric constant (approx. 3–40) compared to free water (approx. 80) under an electromagnetic field. Consequently, a clay soil with high bound water content will yield a lower sensor output than a sandy soil at the same volumetric water content.

Even at the same volumetric water content, a specific sensor’s response varies significantly across different soil types [50,57]. In order to overcome this limitation, early studies have proposed establishing soil moisture content prediction models for different soil types [58,59]. However, this method faces challenges in practical applications, because the targeted calibration work requires high time and labor costs. To this end, researchers have begun to explore common calibration models for multiple soil types to improve the adaptability and measurement consistency of sensors in different soil environments.

In order to reduce the influence of different soil types on the measurement results, research has been devoted to the development of universal calibration models to achieve calibration-free measurements. Deng et al. [60] proposed a calibration-free capacitive soil moisture detection method based on sand content. Their study established a linear relationship between the sixth power of capacitance $C^{6}$ and volumetric water content, θ, expressed as $C^{6}=k\cdot \left(\theta -0.04\right)$. Furthermore, they derived a linear empirical formula linking the slope parameter k to the sand content ω through experimental data fitting: $k=4.07191\times 10^{17}-3.28458\times 10^{17}\cdot \omega$. As a result, they developed a calibration-free model to directly calculate volumetric water content based on capacitance measurements with only prior knowledge of soil sand content. The model’s accuracy was evaluated through three repetitive experiments and compared against the gravimetric method. The results show that the MAME and RMSE of the model are less than 3.8% and 2.0%, respectively. Scudiero et al. [61] developed a universal volumetric water content calibration model for a variety of soils. The model optimizes the traditional logarithmic model by introducing key soil properties such as soil organic carbon and clay/sand ratio, and the RMSE of the model reaches 3.8%.

However, claims regarding “calibration-free” or “universal” models must be critically challenged. In reality, the universality of these generalized approaches is strictly bounded by fundamental physical limits. First, regarding soil structure, universal equations often assume a uniform homogeneous matrix, failing to account for variations in bulk density, field compaction, and porosity. Even within the same soil type, structural degradation or tillage can significantly alter the dielectric bulk volume, introducing severe biases in “universal” predictions. Second, from the perspective of mineralogy, simply categorizing soil by aggregate clay percentage is fundamentally insufficient. Different clay minerals exhibit vastly different specific surface areas and cation exchange capacities (e.g., highly expansive montmorillonite versus non-expansive kaolinite). These mineralogical differences drastically change the bound-to-free water ratio, meaning two soils with identical macroscopic textures can exhibit entirely different dielectric responses. Finally, across different moisture regimes, these generalized models frequently lose accuracy at extreme boundaries. They struggle to capture the complex dielectric transitions in ultra-dry soils (where bound water polarization dominates) and near saturation (where hysteresis effects and structural swelling occur). Therefore, a truly “universal” model remains theoretically elusive. Existing generalized models should be critically viewed as practical approximations constrained to specific regional conditions, rather than globally applicable physical laws.

**Figure 7:**
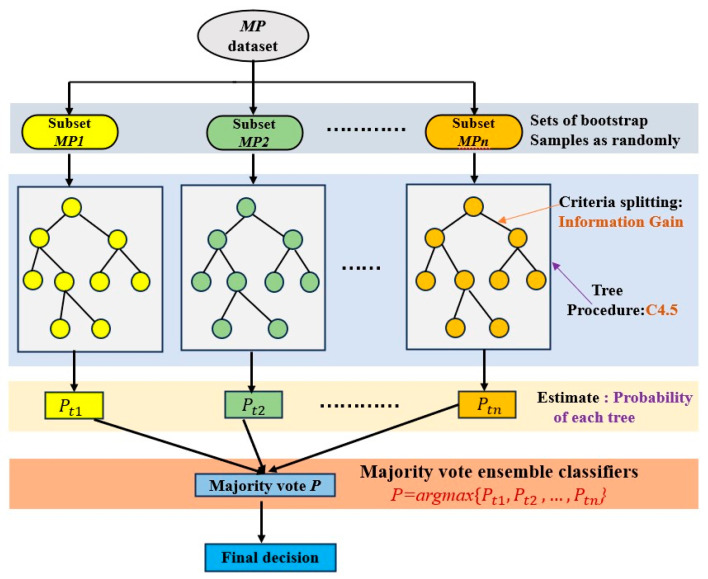
Model of the bagged tree. The original MP dataset is separated into multiple training subsets through bootstrap sampling. Individual decision trees are constructed using the C4.5 algorithm based on these subsets. The final prediction result P is determined by the majority vote of all individual tree outputs ($P_{t1}$ to $P_{tn}$), aimed at reducing prediction variance and avoiding over-fitting. (Adapted from Reference [62]).

Alternatively, an alternative strategy involves first identifying the soil type and then constructing a predictive model accordingly to enhance measurement accuracy. Song et al. [62] proposed a farmland environmental monitoring system based on a data fusion algorithm, which improved the measurement accuracy of the FDR soil moisture sensor by a two-stage calibration method. In the first stage, a linear compensation method is used to calibrate the linear drift caused by sensor aging or corrosion. In the second stage, the soil type was classified by the Bagged Tree model (as shown in Figure 7), and then the soil type, temperature, hardness and the output of the sensor were input into the BP neural network for data fusion. The experimental results show that the MAE, MSE and RMSE of the sensor are reduced by 1.37%, 3.79 and 1.86% respectively after calibration. Malajner et al. [63] employed a Support Vector Machine (SVM) model to classify soil types and selected appropriate moisture–dielectric constant models based on the classification results for calculating soil moisture content. By automatically choosing the most suitable model, the moisture estimation error was significantly reduced, thereby improving measurement accuracy.

Table 5 should likewise be read as a structured summary of soil-type calibration studies rather than a direct comparison table. The main value of this section is to distinguish calibration objectives, validation contexts, and applicability boundaries of generalized and classification-based approaches, instead of ranking them by raw reported metrics obtained from different soil datasets.

**Table 5:** Structured evidence summary of soil-type calibration methods.

Category	Reference	Method	Strategy Description	Metrics (Note: Conditions Vary)
Generalized Models	[60]	Calibration-free	Empirical formula based on sand content	MAME < 3.8%; RMSE < 2.0%
	[61]	Universal Model	Logarithmic model with clay/sand ratio	RMSE:3.8%
Classification-based	[62]	Bagged Tree + BPNN	Two-stage: Classify soil and then fuse data	MAE reduced by 1.37%; RMSE reduced by 1.86%
	[63]	SVM Classification	Auto-selection of moisture models	Error significantly reduced

Note: Table 5 uses the same reporting logic as Table 2, Table 3 and Table 4. Because the included studies differ in soil dataset, texture composition, calibration target, and evaluation metric, the listed values indicate study-specific effectiveness only and are not intended for direct metric-level comparison across all entries.

Table 5 lists the comparisons among different calibration methods for soil types. According to existing studies, calibration approaches addressing the measurement errors of capacitive soil moisture sensors caused by variations in soil type can generally be divided into two main categories. The first involves developing generalized calibration models that can be applied across different soil types to enhance sensor adaptability. Another approach is to first identify the soil type and then select the corresponding moisture content prediction model based on the classification results. In addition, some studies have proposed mitigating soil-type-induced measurement deviations by increasing the operating frequency of the sensor [38,51]. However, excessively high operating frequencies may introduce electromagnetic noise, resulting in unstable sensor outputs. With the advancement of machine learning and data fusion techniques, multi-factor coupled modeling and data-driven adaptive calibration methods have emerged as promising research directions. Future work should focus on improving model generalization and environmental robustness to enhance the long-term stability and applicability of capacitive soil moisture sensors under complex soil conditions. Finally, the current research on soil type calibration is relatively limited, and few studies have considered the joint calibration of temperature, conductivity and soil type. In theory, combining the soil type calibration method with the aforementioned temperature and conductivity calibration method may help to improve the measurement stability and accuracy of the sensor under different environmental conditions, which also provides a direction for future multi-factor joint calibration research.

## 7. Multi-Parameter Synergies Among Temperature, Conductivity, and Soil Type

Although Section 4, Section 5 and Section 6 review temperature, electrical conductivity, and soil type separately for organizational clarity, their effects are physically coupled in real soils and therefore cannot be rigorously corrected by independent single-factor equations. Soil type is not merely an additional categorical variable; it modulates both the thermal response of the dielectric spectrum and the conductivity pathway through differences in clay mineralogy, specific surface area, pore-size distribution, bulk density, and the ratio of bound to free water [43,64]. Consequently, the same increase in temperature or salinity can produce markedly different apparent-permittivity shifts in coarse-textured and fine-textured soils.

This coupling can be understood from the perspective of complex permittivity. Temperature changes alter molecular relaxation and ion mobility, and conductivity mainly contributes to dielectric loss; soil type governs how water is partitioned and how conductive paths are formed. Because these processes act simultaneously, the sensor output is a nonlinear superposition of texture-dependent polarization, temperature-dependent relaxation, and salinity-related conduction loss, rather than the sum of three separable disturbances [42,65]. This also explains why models calibrated under a single soil class or narrow environmental window often degrade when transferred to soils with different textures or salinity backgrounds.

Therefore, the main scientific bottleneck is not only improving the correction accuracy for each variable separately but establishing calibration strategies that explicitly incorporate their joint action. In practical terms, future FDR calibration should move toward multi-input frameworks that combine temperature, bulk or pore-water conductivity, and soil-texture descriptors or soil-class information within a unified model, preferably constrained by dielectric physics rather than purely empirical fitting [17,51]. Such an integrated perspective better explains the limitations of current single-factor studies and clarifies why holistic multi-parameter decoupling is necessary for robust field deployment. Direct studies explicitly addressing the full three-way coupling of temperature, conductivity, and soil type in FDR-based capacitive soil moisture sensors remain limited; accordingly, this section is intended as an integrative synthesis of the available evidence rather than as a summary of an already mature three-factor calibration literature.

## 8. Existing Problems and Prospects

While various calibration methods have demonstrated significant progress, a critical evaluation reveals distinct trade-offs in robustness, scalability, and field applicability, as summarized in Table 6. More importantly, the reviewed literature already points to three evidence-based limitations that motivate the future research agenda. First, most conductivity and multi-parameter studies still rely on empirical compensation within limited soil classes and environmental ranges, rather than on explicit physical separation of coupled interference. Second, many machine-learning models achieve strong within-study accuracy but are trained under relatively homogeneous conditions, which raises concerns about cross-site transferability and retraining cost. Third, the models with the strongest nonlinear fitting ability usually impose the highest computational burden, which directly constrains deployment in low-power field nodes.

**Table 6:** Critical evaluation of calibration strategies based on robustness, scalability, and field applicability.

Calibration Strategy	Robustness (Handling Non-linearity)	Scalability (Deployment Complexity)	Field Applicability	Overall Critical Evaluation
Hardware Compensation (e.g., Differential Capacitance, Bridge Circuit)	Low to Medium	Low once designed	High	Best for low-cost, large-scale deployments where stability is prioritized over extreme precision.
Conventional Regression (e.g., Polynomial and Linear)	Medium	Low	Medium	The standard “middle-ground” choice. Reliable for stable environments but fails in extreme soil conditions.
Machine Learning Models (e.g., BPNN, RF, XGBoost)	High	High	Medium	The gold standard for high-precision research. Currently, it is less practical for mass commercial deployment due to data and hardware costs.

Furthermore, the reviewed literature is not balanced across the three calibration targets. As shown by the evidence summarized in Section 4, Section 5 and Section 6 and Table 2, Table 3, Table 4 and Table 5, temperature calibration has been investigated more extensively, whereas conductivity calibration, coupled temperature-conductivity correction, and soil-type calibration remain comparatively less and more condition-specific. This imbalance is not simply a matter of research attention; it also reflects the increasing physical difficulty of isolating different components of the dielectric response under realistic field conditions.

For electrical conductivity, the key difficulty identified in the reviewed studies is conductive loss associated with ionic conduction. Under high salinity conditions, increased electrical conductivity contributes to the imaginary component of the complex permittivity ($\varepsilon_{r}^{\prime \prime }$), which reduces the Q-factor of the resonant circuit and attenuates the signal amplitude. As a result, the measured capacitance becomes intrinsically biased by conductive loss rather than reflecting only the real permittivity component ($\varepsilon_{r}^{\prime }$). This helps explain why the conductivity-related studies reviewed in Section 5 often require explicit correction models and why simple frequency adjustment alone is not sufficient for complete compensation.

For soil type, the challenge is even more fundamental. Soil is an extremely heterogeneous multiphase system, and its “texture” cannot be parameterized as a simple continuous variable. Variations in specific surface area, clay mineralogy, pore structure, and organic matter content strongly influence the proportion of bound versus free water and thereby alter dielectric response characteristics. This is consistent with the studies reviewed in Section 6, where calibration performance remains highly dependent on soil class, generalized models are difficult to construct, and many approaches still trade universality for local accuracy.

In light of these challenges, the future development of FDR-based sensors must move beyond simple accuracy improvements. To genuinely advance the field, researchers must prioritize and resolve the following three key scientific bottlenecks:

Bottleneck 1: Physical Decoupling of Multi-Parameter Interference under Complex Conditions. The literature synthesized in Section 5.2, Section 6 and Section 7 shows that current multi-factor models still mainly compensate coupled effects through empirical fitting, while only a limited number of studies explicitly resolve how temperature, conductivity, and soil type jointly alter the measured dielectric response. This limitation becomes especially important when boundary conditions change sharply, such as in saline soils, freeze–thaw environments, or soils with strong textural heterogeneity. Therefore, the key next step is to move from variable addition toward physically interpretable decoupling of bound-water effects, ionic conduction, and texture-dependent polarization.

Bottleneck 2: Generalization and Cross-Site Transferability of Data-Driven Calibration. As discussed in Section 4.2.2 and reflected in the comparative synthesis of Table 2, Table 4, Table 5 and Table 6, machine-learning models often deliver excellent accuracy within the datasets on which they are trained, but their robustness across soil types, sites, and environmental ranges remains insufficiently validated. This means that the current bottleneck is not only model accuracy, but also whether the learned relationship is transferable beyond the calibration domain. Accordingly, future work should place greater emphasis on dataset diversity, physically meaningful inputs, external validation, and adaptive strategies such as transfer learning, rather than assuming that higher in-sample accuracy alone will guarantee field reliability.

Bottleneck 3: Deployment Feasibility of Advanced Models on Resource-Constrained Hardware. The comparative discussion in Section 8 and Table 6 shows a clear tension between nonlinear modeling capability and deployment burden: the most flexible models are often also the most computationally demanding. This is why Edge-AI should be viewed not as an isolated future concept, but as a bottleneck that emerges directly from the reviewed literature. If real-time or online calibration is to be implemented in battery-powered IoT nodes, future studies must evaluate not only prediction accuracy but also memory footprint, inference latency, energy consumption, and model-compression feasibility.

## Figures and Tables

**Table 1:** The dielectric constant of the main components of soil. (According to Reference [12]).

Composition	Quartz	Air	Dry Soil Minerals	Water	Clay Minerals	Ice
dielectric constant	3.8	1	2~5	78.2	5~40	3

**Table 2:** Structured evidence summary of temperature-calibration methods.

Category	Reference	Method	Key Performance/Effect	Experimental Metrics (Note: Conditions Vary)
Hardware Compensation	[19]	Differential Capacitance Technique	Drift reduced from 22.5 fF/°C to 4.9 fF/°C	
	[20]	Bridge Circuit	Temp-induced error reduced from 22.4% to 1.24%	Accuracy: 98.76%
Software: Regression	[22]	Polynomial regression	-	$R^{2}:\;0.9931$
Software: Machine Learning	[27]	BPNN	Readings aligned with gravimetric method	-
	[28]	SVM	High stability in dynamic tests	$R^{2}:\;0.91$; Max Error: 4.62%
	[29]	XGBoost	Superior to RF, KNN, and SVR in test set	$R^{2}:\;0.973$; RMSE: 1.165%
	[30]	Gaussian process regression	-	RMSE: 0.78%; MAE: 0.44%
	[30]	Multivariate adaptive regression splines	-	RMSE: 1.02%; MAE: 0.65%
Software: Metaheuristic Algorithms	[32]	GA-BPNN	Significant error reduction over standard BP	MSE: $\;1.9683\times 10^{-5}$
	[33]	WOA-BPNN	Improved stability over SVM/RF	Accuracy: 98.8%; $\mathrm{MSE}:\;5.13\times 10^{-3}$
	[34]	Logistic-SSA-ELM	Overcame local optima issues	$R^{2}:\;0.7016$; RMSEP: 3.715

Note: Table 2 includes both direct soil-based studies and a limited number of cross-domain studies used only as methodological references for temperature compensation. These studies share a similar capacitive/FDR-based measurement principle, but may differ in measured medium, sensor configuration, temperature range, and validation protocol. Therefore, the reported metrics are provided to summarize within-study effectiveness and transferable calibration logic, rather than to support direct quantitative comparison across all studies. “-” indicates data not reported in the original source.

## Data Availability

The raw data supporting the conclusions of this article will be made available by the authors upon request.

## References

[1] Zhang X., Feng G., Sun X. (2024). Advanced technologies of soil moisture monitoring in precision agriculture: A Review. J. Agric. Food Res..

[2] Xu Y., Chen Q., Hu D., Li B., Tang H. (2022). Modeling and Testing of Fringe-Field Capacitive Moisture Sensor Under Certain Electrode Area. IEEE Trans. Instrum. Meas..

[3] Hao P., Han J., Liu X., Song L., Miao F., Feng J. (2024). Technical status and development trend of soil moisture content measurement methods. Jiangsu Agric. Sci..

[4] Dou J., Guo Y., Wang S., Liu J. (2017). Study on Determination Methods of Moisture Content in Soil. J. Shanxi Agric. Sci..

[5] Li Y., Wang Y., Huang Z. (2017). Study on Probe-type Soil Moisture Sensor. Instrum. Technol..

[6] Alhadchiti A., Nikolic B., Ioakim P., Powner M.B., Triantis I.F. (2025). Identification and analysis of key factors limiting the performance of electrical soil sensors: A review. Comput. Electron. Agric..

[7] Ivanova M., Georgiev G., Dimitrova R., Rangelov Y. Methods for Measurement of Electrical Characteristics of Soils. A Review. Proceedings of the 2024 16th Electrical Engineering Faculty Conference (BulEF).

[8] Abdulraheem M.I., Chen H., Li L., Moshood A.Y., Zhang W., Xiong Y., Zhang Y., Taiwo L.B., Farooque A.A., Hu J. (2024). Recent Advances in Dielectric Properties-Based Soil Water Content Measurements. Remote Sens..

[9] Adla S., Bruckmaier F., Arias-Rodriguez L.F., Tripathi S., Pande S., Disse M. (2024). Impact of calibrating a low-cost capacitance-based soil moisture sensor on AquaCrop model performance. J. Environ. Manag..

[10] Adla S., Rai N.K., Sri Karumanchi H., Tripathi S., Disse M., Pande S. (2020). Laboratory Calibration and Performance Evaluation of Low-Cost Capacitive and Very Low-Cost Resistive Soil Moisture Sensors. Sensors.

[11] Xu J., Ma X., Logsdon S.D., Horton R. (2012). Short, Multineedle Frequency Domain Reflectometry Sensor Suitable for Measuring Soil Water Content. Soil Sci. Soc. Am. J..

[12] Qian H. (2017). Design and Implementation of Portable Soil Profile Moisture Sensor. Master’s Thesis.

[13] Hu J., Li L., Iderawumi A.M., Yuan F., Li B., Wei W. (2021). Research progress of soil water content measurements using dielectric properties. J. Henan Agric. Univ..

[14] Xu Y., He Y., Li X., Tu Y., Zhang K., Liu Y., Sun Y. (2026). Novel Spiral and Embracing IDE Capacitive Sensors for In Situ Measurement of Soil Moisture. Sensors.

[15] Qian L. (2016). Research on Soil Moisture Detection Device Based on FDR Technology. Master’s Thesis.

[16] Topp G.C., Davis J.L., Annan A.P. (2010). Electromagnetic determination of soil water content: Measurements in coaxial transmission lines. Water Resour. Res..

[17] Mane S., Das N., Singh G., Cosh M., Dong Y. (2024). Advancements in dielectric soil moisture sensor Calibration: A comprehensive review of methods and techniques. Comput. Electron. Agric..

[18] Yu L., Gao W., R. Shamshiri R., Tao S., Ren Y., Zhang Y., Su G. (2021). Review of research progress on soil moisture sensor technology. Int. J. Agric. Biol. Eng..

[19] Mohamed D., Saari M.M., Ismail A. Evaluation of Differential Capacitance Technique in LC Resonant-Based Capacitance Sensor for Moisture Content Detection in Paddy Seeds. Proceedings of the 2024 International Conference on System Science and Engineering (ICSSE).

[20] Javed M., Sajid M., Yousaf H.M.Z., Hassan G., Mahmood H. (2021). Facile and Low Cost Temperature Compensated Humidity Sensor and Signal Conditioning System. IEEE Sens. J..

[21] Cao M., Xu X., Su Y., Song T., Zhou M., Jia W. (2015). Research on Temperature Effect on FDR Soil Moisture Sensor. Water Sav. Irrig..

[22] Yang W., Wang Y., Yao Y., Sai J. (2019). Soil Moisture Monitoring System Based on Narrow Band Internet of Things. Trans. Chin. Soc. Agric. Mach..

[23] Zhang J., Xie S., Liu J., Chen C., Zhao L. (2018). Research on the Temperature Compensation Model of the FDR Soil Moisture Sensor. J. Agric. Mech. Res..

[24] Xu W., Feng X., Xing H. (2019). Modeling and Analysis of Adaptive Temperature Compensation for Humidity Sensors. Electronics.

[25] Xing H., Peng J., Lü W., Xu W., Wu X. (2012). A Fusion Algorithm for Humidity Sensor Temperature Compensation. Chin. J. Sens. Actuators.

[26] Mahaseth D.N., Kumar L., Islam T. (2018). An efficient signal conditioning circuit to piecewise linearizing the response characteristic of highly nonlinear sensors. Sens. Actuators A Phys..

[27] Gnecchi J.A.G., Tirado L.F., Campos G.M.C., Ramirez R.D., Gordillo C.F.E. Design of a Soil Moisture Sensor with Temperature Compensation Using a Backpropagation Neural Network. Proceedings of the 2008 Electronics, Robotics and Automotive Mechanics Conference.

[28] Han C., Wang Y., Shi Z., Xu Y., Qiu S., Mao H. (2024). The Design and Experimentation of a Corn Moisture Detection Device Based on Double Capacitors. Sensors.

[29] Shen X., Wu Y., Meng F., Zhang G., Yu J., Shi K. (2021). Research on Temperature Compensation Model for Soil Moisture Content Sensors Based XGBoost. Water Sav. Irrig..

[30] Chen L., Zhangzhong L., Zheng W., Yu J., Wang Z., Wang L., Huang C. (2019). Data-Driven Calibration of Soil Moisture Sensor Considering Impacts of Temperature: A Case Study on FDR Sensors. Sensors.

[31] Yan F., Lin Z., Wang X., Azarmi F., Sobolev K. (2017). Evaluation and prediction of bond strength of GFRP-bar reinforced concrete using artificial neural network optimized with genetic algorithm. Compos. Struct..

[32] Sun W., Wan L., Che G., Xu P., Wang H., Qu T. (2023). Design and Experiment of Capacitive Rice Online Moisture Detection Device. Sensors.

[33] Jiang J., Xu G., Wang H., Yang Z., Sun B., Guan C., Feng J., Ma Y., Chen X. (2024). High-accuracy road surface condition detection through multi-sensor information fusion based on WOA-BP neural network. Sens. Actuators A Phys..

[34] Qiu Z., Li G., Huang Z., He X., Zhang Z., Li Z., Du H. (2024). Research on non-destructive and rapid detection technology of foxtail millet moisture content based on capacitance method and Logistic-SSA-ELM modelling. Front. Plant Sci..

[35] Dang R., Zhang H., Song N., Dang B., Wang D. (2016). Compensation and calibration of the high temperature and pressure downhole pressure sensor. Chin. J. Sci. Instrum..

[36] Kargas G., Soulis K.X. (2019). Performance evaluation of a recently developed soil water content, dielectric permittivity, and bulk electrical conductivity electromagnetic sensor. Agric. Water Manag..

[37] Diekmann A. (2023). Soil moisture sensing in saltwater: A review. Environ. Earth Sci..

[38] Deng X., Gu H., Yang L., Lyu H., Cheng Y., Pan L., Fu Z., Cui L., Zhang L. (2020). A method of electrical conductivity compensation in a low-cost soil moisture sensing measurement based on capacitance. Measurement.

[39] Zhang X., Xie F., Sheng Q., Ni M., Zhang J., Xu Y. (2024). Calibration Method of Soil Moisture Capacitance Sensor Based on Compensation of Electrical Conductivity to Relative Permittivity. Trans. Chin. Soc. Agric. Mach..

[40] Santos C.P.d., Rodrigues A.A., Canafístula F.J.F., Rocha Neto O.C.d., Daher S., Teixeira A.d.S. (2022). Performance of the capacitive moisture sensor under different saline conditions. Rev. CiÊncia AgronÔmica.

[41] Szypłowska A., Lewandowski A., Kafarski M., Szerement J., Wilczek A., Budzeń M., Majcher J., Skierucha W. (2023). Influence of Temperature on Soil Dielectric Spectra in the 20 MHz–3 GHz Frequency Range. IEEE Trans. Geosci. Remote Sens..

[42] Ko H., Choo H., Ji K. (2023). Effect of temperature on electrical conductivity of soils—Role of surface conduction. Eng. Geol..

[43] Liu L., Li W., Lu Y., Ren T., Horton R. (2023). Relationship between thermal and electrical conductivity curves of soils with a unimodal pore size distribution: Part 1. A unified series-parallel resistor model. Geoderma.

[44] Chandel A., Swami D., Joshi N. (2024). Calibration complexities: Full-scale error impact and simultaneous variation of salinity, temperature, and moisture content on sensor performance in soil. Environ. Dev. Sustain..

[45] Gu H., Yang L., Deng X., Lü H., Song Z., Pan L., Zhang L., Cui L. (2020). Research on Multi-parameter Calibration Method for Capacitive Soil Moisture Detection. Instrum. Tech. Sens..

[46] Bogena H.R., Huisman J.A., Oberdörster C., Vereecken H. (2007). Evaluation of a low-cost soil water content sensor for wireless network applications. J. Hydrol..

[47] Wang Z., Hu S., Ma R., Sun Z., Ge M., Wang J., Qiao S. (2023). Calibration of capacitive soil moisture sensor based on random forest. Bull. Geol. Sci. Technol..

[48] Moghadas D., Badorreck A. (2019). Machine learning to estimate soil moisture from geophysical measurements of electrical conductivity. Near Surf. Geophys..

[49] Rhoades J.D., Raats P.A.C., Prather R.J. (1976). Effects of Liquid-phase Electrical Conductivity, Water Content, and Surface Conductivity on Bulk Soil Electrical Conductivity. Soil Sci. Soc. Am. J..

[50] Cappelli I., Parri L., Bichi B., Mugnaini M., Vignoli V., Fort A. Low-Cost Sensors for Soil Moisture Measurement: Modeling and Characterization. Proceedings of the 2023 IEEE International Workshop on Metrology for Agriculture and Forestry (MetroAgriFor).

[51] Szypłowska A., Lewandowski A., Jones S.B., Sabouroux P., Szerement J., Kafarski M., Wilczek A., Skierucha W. (2019). Impact of soil salinity, texture and measurement frequency on the relations between soil moisture and 20 MHz–3 GHz dielectric permittivity spectrum for soils of medium texture. J. Hydrol..

[52] Aranda Britez D.A., Tapia A., Millán Gata P. (2025). A self-calibration algorithm for soil moisture sensors using deep learning. Appl. Intell..

[53] Abdelmoneim A.A., Al Kalaany C.M., Dragonetti G., Derardja B., Khadra R. (2025). Comparative Analysis of Soil Moisture- and Weather-Based Irrigation Scheduling for Drip-Irrigated Lettuce Using Low-Cost Internet of Things Capacitive Sensors. Sensors.

[54] Marino P., Roman Quintero D.C., Santonastaso G.F., Greco R. (2023). Prototype of an IoT-Based Low-Cost Sensor Network for the Hydrological Monitoring of Landslide-Prone Areas. Sensors.

[55] Jamroen C., Komkum P., Fongkerd C., Krongpha W. (2020). An Intelligent Irrigation Scheduling System Using Low-Cost Wireless Sensor Network Toward Sustainable and Precision Agriculture. IEEE Access.

[56] Dey A., Sharma C., Sarma U. Fringing Field Capacitive Sensor: A Study of Different Configurations for Soil Moisture Measurement. Proceedings of the 2022 International Interdisciplinary Conference on Mathematics, Engineering and Science (MESIICON).

[57] Deng C., Li Y., Liu M., Lin W., Su N. (2017). Research on different types of soil moisture using FDR. Water Resour. Informatiz..

[58] Dafonte J., Gonzalez M.A., Comesana E., Teijeiro M.T., Cancela J.J. (2024). Soil Water Status Monitoring System with Proximal Low-Cost Sensors and LoRa Technology for Smart Water Irrigation in Woody Crops. Sensors.

[59] Matula S., Batkova K., Legese W.L. (2016). Laboratory Performance of Five Selected Soil Moisture Sensors Applying Factory and Own Calibration Equations for Two Soil Media of Different Bulk Density and Salinity Levels. Sensors.

[60] Deng X., Yang L., Fu Z., Du C., Lyu H., Cui L., Zhang L., Zhang J., Jia B. (2021). A calibration-free capacitive moisture detection method for multiple soil environments. Measurement.

[61] Scudiero E., Berti A., Teatini P., Morari F. (2012). Simultaneous monitoring of soil water content and salinity with a low-cost capacitance-resistance probe. Sensors.

[62] Song T., Si Y., Gao J., Wang W., Nie C., Klemeš J.J. (2023). Prediction and monitoring model for farmland environmental system using soil sensor and neural network algorithm. Open Phys..

[63] Malajner M., Gleich D., Planinsic P. (2019). Soil type characterization for moisture estimation using machine learning and UWB-Time of Flight measurements. Measurement.

[64] Bi Y., Cui B., Li S., Fan N., Gu T., Feng R. (2026). Study on the combined influence mechanism of temperature, density and moisture content on the resistivity of loess. Sci. Rep..

[65] Schillaci C., Scarpa S., Yunta F., Lipani A., Visconti F., Szatmári G., Balog K., Koganti T., Greve M., Bondi G. (2025). Empirical estimation of saturated soil-paste electrical conductivity in the EU using pedotransfer functions and Quantile Regression Forests: A mapping approach based on LUCAS topsoil data. Geoderma.

